# Evaluating the effect of healthcare providers on the clinical path of heart failure patients through a semi-Markov, multi-state model

**DOI:** 10.1186/s12913-020-05294-3

**Published:** 2020-06-12

**Authors:** Francesca Gasperoni, Francesca Ieva, Anna Maria Paganoni, Christopher H Jackson, Linda Sharples

**Affiliations:** 1 MRC Biostatistics Unit, University of Cambridge, Forvie Site, Robinson Way, Cambridge, CB2 0SR UK; 2 MOX laboratory, Department of Mathematics, Politecnico di Milano, Piazza Leonardo da Vinci, 32, Milan, 20133 Italy; 3 CADS-Center for Analysis, Decisions and Society, Human Technopole, Via Cristina Belgioioso, 171, Milan, 20157 Italy; 4 CHRP-National Center for Healthcare Research and Pharmacoepidemiology, University of Milano-Bicocca, Via Bicocca degli Arcimboldi, 8, Milan, 20126 Italy; 5 Department of Medical Statistics, London School of Hygiene & Tropical Medicine, Keppel Street, London, WC1E 7HT UK

**Keywords:** Clustering, Decision making, Multi-state model, Nonparametric frailty

## Abstract

**Background:**

Investigating similarities and differences among healthcare providers, on the basis of patient healthcare experience, is of interest for policy making. Availability of high quality, routine health databases allows a more detailed analysis of performance across multiple outcomes, but requires appropriate statistical methodology.

**Methods:**

Motivated by analysis of a clinical administrative database of 42,871 Heart Failure patients, we develop a semi-Markov, illness-death, multi-state model of repeated admissions to hospital, subsequent discharge and death. Transition times between these health states each have a flexible baseline hazard, with proportional hazards for patient characteristics (case-mix adjustment) and a discrete distribution for frailty terms representing clusters of providers. Models were estimated using an Expectation-Maximization algorithm and the number of clusters was based on the Bayesian Information Criterion.

**Results:**

We are able to identify clusters of providers for each transition, via the inclusion of a nonparametric discrete frailty. Specifically, we detect 5 latent populations (clusters of providers) for the discharge transition, 3 for the in-hospital to death transition and 4 for the readmission transition. Out of hospital death rates are similar across all providers in this dataset. Adjusting for case-mix, we could detect those providers that show extreme behaviour patterns across different transitions (readmission, discharge and death).

**Conclusions:**

The proposed statistical method incorporates both multiple time-to-event outcomes and identification of clusters of providers with extreme behaviour simultaneously. In this way, the whole patient pathway can be considered, which should help healthcare managers to make a more comprehensive assessment of performance.

## Background

Evaluation of public services, such as hospitals, nursing homes and intermediate care units is widespread and encompasses researchers from several disciplines, including clinical epidemiology, statistics and economics. Interest focuses on identifying providers with results that are extreme, in that overall performance lies in the tails of the distribution of results for the sample of providers in the study.

There are two main approaches to benchmarking healthcare providers. First, internal benchmarking involves setting a fixed target for outcomes and assessing providers’ results against those targets; an example might be the comparison of observed 30-day post-operative survival against a fixed target of, say 95%. Surgical units that fail to achieve this target may be investigated further to identify systematic factors causing the poor performance. Alternatively, in competitive benchmarking, the results for all providers are analysed together, and those providers with extreme results subjected to more detailed investigation. In this paper, interest is in the situation where all providers are assessed together (competitive bench-marking), although it is important to stress that we are not interested in ranking providers, which is a highly data-dependent process and can lead to unsafe policy decisions (like star-ratings) [1]. Rather, our interest is in exploration of differences in patient experience of healthcare in order to identify groups of providers for further investigation.

Within this framework it is not clear how to identify groups of healthcare providers for further study. One option is set an *a priori* proportion for further study, for example the 5% of all providers with the lowest performance by some measure. However, such an *ad hoc* decision threshold may result in some poor performance being missed or some adequate performance being classified as poor. It is also highly dependent on the tail behaviour of the distribution of provider outcomes. A more principled method for identifying groups of providers with similar but extreme outcomes would be useful.

Statistical approaches for evaluating providers depend on availability of an objective and universally-recorded outcome measure, such as 30-day mortality, duration of time spent in hospital and major clinical events (for example, heart attacks or strokes) [2, 3]. The most common statistical approach for comparing providers is to fit logistic regression or hierarchical logistic regression models for a single binary outcome [4]. Whilst this is a useful methodology if interest is in a single short-term event, and can provide easily understood summaries of provider outcomes, it is inefficient for identifying differences in longer-term outcomes, such as time to clinical events, including time to death, time to hospital readmission and time to discharge. Time-to-event outcomes provide a more detailed and sensitive characterisation of both (case-mix adjusted) provider performance and patient clinical history. One limitation of the analysis of time-to-event outcomes is that they require individual patient data, whilst published studies in this context have often been based on aggregated information, such as the percentage of in-hospital deaths, the average Length-of-Stay (LOS) and average waiting times. However, the increased availability of high quality, routinely-recorded healthcare data has opened up the possibility of a deeper analysis of provider performance, based on individual patient outcomes.

Both increasing average age and the associated prevalence of multiple chronic conditions in contemporary patient populations can complicate the assessment of healthcare management, so that methods that can accommodate multi-dimensional outcomes would be useful. However, we are aware of only one study that has provided a strategy to rank providers in a multi-dimensional setting, by proposing a dominance criterion [5]. Thus, methods for assessing multiple healthcare outcomes are required.

In this work we propose a novel methodology to address these three main issues,

(i) identification of groups of providers for further study,
(ii) using multiple outcomes,
(iii) arising from time-to-event observations.

Specifically, we introduce a statistical model of hospital admissions and death, based on a semi-Markov (state-transition) model, together with semi-parametric, mixed-effects survival models, with discrete random effects for providers, for each transition intensity between states. This assumes that providers can be grouped according to the similarity in their distribution of times at which their patients move between health states in the model. Multiple outcomes of interest are represented by different states in the model. Groups with high or low rates of particular transitions between states can be studied further, and providers might be grouped in different ways according to different outcomes. The approach is novel in that it assesses providers by considering the whole clinical history of their patients and, by focusing on groups of providers with common random effects, it avoids *ad hoc* selection criteria for providers. The decision-making method that we propose is particularly suitable for exploring provider outcomes using large clinical, research and administrative databases. In such cases, it is difficult to select *a priori* a specific set of criteria by which to assess provider performance, or to weight criteria [6].

The combination of a multi-state model with discrete frailty parameters has not been explored yet in the statistical literature. The frailty parameter, or random effect, was introduced for the first time in the context of survival analysis by Vaupel et al. [7], in order to capture heterogeneity among patients or group of patients that could not be explained by the available covariates. In a time-to-event model, patients or groups with the same frailty are assumed to have the same rate of experiencing the event, after adjusting for observed covariates. In survival analysis, the event is death, hence the term *frailty*, but note this term may also be used to refer to the rate of any kind of event. The concept of frailty is analogous to the random effect in linear and generalised linear mixed effect models. Few researchers have proposed multi-state models with frailties for dealing with multiple time-to-event data [8–13]. With the exception of the joint frailty model proposed by Liquet et al. [11], most published articles have used frequentist approaches and assumed either: parametric frailties that differed between transitions or models that include a Markov or semi-Markov assumption. A hierarchical Bayesian model has also been introduced [8]. In these studies, assumed frailty distributions were usually Gamma [9–11], log-Normal [13] or the Compound Poisson [12], and establishing the best frailty distribution is not straightforward in this context [14]. Moreover, the focus of these studies was the relationship between provider characteristics as covariates and the rate of transition between health states, rather than the provider effects themselves. In our approach, we relax the strict *a priori* assumption on the frailty distribution to allow a nonparametric discrete frailty, which has previously only been done for times to single events [15] rather than multi-state models.

This work was motivated by the need to explore (case-mix adjusted) outcomes using data from a clinical administrative database of Heart Failure (HF) patients, hospitalised in the Lombardia Region of Italy. HF is typical of chronic syndromes affecting elderly populations, and confers high risk of hospital readmission and death; both outcomes are clinically important and indicative of provider performance [16]. The work builds on a small number of previous studies that have provided a more complete analysis of the clinical pathway of HF patients [17–20].

The paper is organised as follows: in “Methods” section we present the dataset that motivates this work, describe the proposed statistical methodology and discuss some computational aspects, in “Results” section the results for the HF application are presented and in “Discussion” section we discuss the novelty of the contribution, some open questions and areas for future work.

## Methods

### Data

Our motivation arises from analysis of a cohort of HF patients identified from a clinical administrative database, collected in the Lombardia Region of Italy. We identified 338,861 admission records for a total of 210,917 patients with a first diagnosis of HF, according to ICD-9-CM, between 2005 and 2012.

Since we are interested in studying the readmission-discharge dynamic, with a specific focus on the hierarchical structure of the data, we selected only those patients who were hospitalised in the same institution throughout and were first discharged with a HF diagnosis between 2006 and 2007. These two selection criteria allowed us to focus on the impact of healthcare providers on patients’ clinical history and to have a more complete view of patients’ healthcare resource use. Then, we restrict analysis to those healthcare providers that contributed at least 20 patients to the dataset, to allow stable estimation of results (this choice is supported by previous simulation studies [15]).

After these exclusions, a cohort of 42,871 HF patients, grouped in 140 hospitals, contributed to the analysis. For the selected cohort we had 5 years of follow-up.

A brief summary of the cohort is reported in Table 1. Columns one, two, three, four and five report descriptive statistics related to those patients who were discharged at least once, twice, three times, four times and five times respectively. Mean (standard deviation) and the total number (percentage) are reported for continuous and categorical variables, respectively. We focussed only on the first five hospital admissions, since only 1.17% of the cohort had more than five admissions. Note that the whole cohort was discharged after the first hospitalisation, so that 42,871 patients contributed to the first column of Table 1. A second discharge was recorded only for those patients who were hospitalised at least twice, specifically 11,305 patients (second column of Table 1). This number can be obtained by subtracting the in-hospital deaths from admission one (4,766), deaths occurring after the first discharge (14,569) and censored patients after the first discharge (12,231) from the whole cohort. The average (standard deviation) age of the cohort at the first hospitalisation was 76.79 (11.69) years and 21,876 (51.03%) were female. 19,269 (44.95%) patients had 3 or more comorbidities and 6,096 (15.8%) underwent one or more procedures. There were 7,015 (16.36%) in-hospital deaths and 19,498 (45.48%) deaths outside hospital during follow-up.

**Table 1:** Descriptive summaries of the cohort according to the admission index. S.D. stands for standard deviation. Repeated admission and discharge transitions were observed for the same subject. All patients were hospitalized at least once: summing 38,105 (discharged) and 4,766 (in-hospital deaths) we obtain 42,871 (see the first column)

	1^*st*^ hosp.	2^*nd*^ hosp.	3^*rd*^ hosp.	4^*th*^ hosp.	5^*th*^ hosp.
patients	42,871	11,305	4,318	1,914	929
discharge (%)	38,105 (88.88)	10,025 (88.68)	3,821 (88.49)	1,687 (88.14)	827 (89.02)
in-hosp. death (%)	4,766 (11.12)	1,280 (11.32)	497 (11.51)	227 (11.86)	102 (10.98)
death after disch. (%)	14,569 (33.98)	3,122 (27.62)	1,069 (24.76)	385 (20.11)	172 (18.51)
censoring after disch. (%)	12,231 (28.53)	2,585 (22.87)	838 (19.41)	373 (19.49)	154 (16.58)
Mean LOS (S.D.)	13.95 (15.59)	12.81 (13.32)	13.07 (12.64)	13.28 (12.57)	14.07 (14.11)
Mean age (S.D.)	76.79 (11.69)	78.28 (10.71)	78.56 (10.31)	78.46 (10.02)	78.35 (9.97)
female (%)	21,876 (51.03)	5,416 (47.91)	1,940 (44.93)	811 (42.37)	378 (40.69)
≥3 comorb.^1^ (%)	19,269 (44.95)	7,535 (66.65)	3,345 (77.47)	1,611 (84.17)	819 (88.16)
# procedures^2^					
0 procedures	36,096 (84.2)	9,617 (85.07)	3,740 (86.61)	1,709 (89.29)	851 (91.6)
1 procedure	2,546 (5.94)	795 (7.03)	321 (7.43)	136 (7.11)	51 (5.49)
2 procedures	3,149 (7.35)	783 (6.93)	226 (5.23)	64 (3.34)	27 (2.91)
3 procedures	1,012 (2.36)	109 (0.96)	31 (0.72)	5 (0.26)	
4 procedures	67 (0.16)	1 (0.01%)			
5 procedures	1 (<10^−3^)				

^1^The considered comorbidities are: metastatic cancer, congestive heart failure, dementia, renal failure, weight loss, hemiplegia, alcohol abuse, malignant tumor, arrhythmia, chronic pulmonary obstructive disease, coagulopathy, complicated diabetes, anemia, fluid and electrolyte disorders, liver disease, peripheral vascular disease, psychosis, pulmonary circulation disorder, HIV status and hypertension.

^2^The considered procedures are: Coronary Artery Bypass Surgery, Percutaneous Transluminal Coronary Angioplasty, Implantable Cardioverter-Defibrillator, Cardiovascular surgery and surgery of other types.

The percentage of those admitted who were women tended to decrease as the admission index increased, while the percentages of people with more than three co-morbidities and with no procedures tended to increase. There was no clear pattern for LOS through time and the percentage of in-hospital deaths seemed to be stable across readmissions.

### Statistical model

**Fig. 1:**
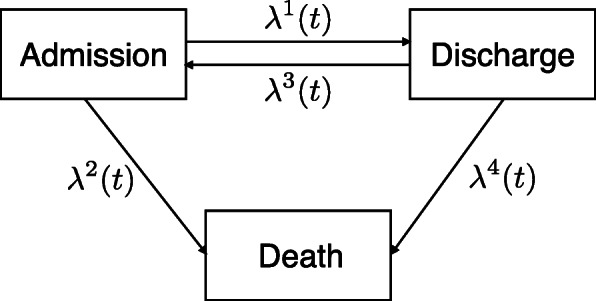
Multi-state model. The proposed multi-state model for the application to clinical administrative data is showed
- it is likely that the hazard is related to time since entry to the current state (while in a Markov model the hazard is related only to the current state and the time of entry to the study).

In order to describe healthcare providers’ effects on the clinical path of HF patients, we propose a semi-Markov (clock reset model), reversible, multi-state model as in Fig. 1. The semi-Markov model was considered appropriate because:

- we know the exact admission, discharge and death times for each patient;

Three quantities are necessary for defining a multi-state model: the state space, the transition-specific hazard rates and the probability of each state occupancy at the time origin (study entry).

The state space *S* is composed of three states (alive in hospital, alive and out of hospital and dead) and there are four possible transitions between these states. Patients may pass between in-hospital and out of hospital states multiple times. Note that the dead state is absorbing in that, once entered, there is no exit transition.

Each transition time is modelled through a recently proposed extension to the Cox proportional hazard model, in which patterns of transition between states exhibit clustering, represented by shared frailty terms that have a discrete distribution [15]. This kind of frailty was first proposed in the simpler framework of time to a single event; it avoids any *a priori* specification of the shape of the frailty distribution and allows detection of clusters of groups (clusters of healthcare providers). We define the detected clusters as *latent populations*, because they represent a latent level of hierarchy in the data, that cannot be explained through the available covariates. Members of the same latent populations share unmeasured characteristics.

In a multi-state framework, we define $\widetilde {T}_{ij}^{l}$ as the time of transition type *l* (*l*=1,...,*L*) for subject *i* (*i*=1,...,*n*_*j*_) in group *j* (*j*=1,...,*J*), while $C_{ij}^{l}$ is the corresponding censoring time. Let $\boldsymbol {X}_{ij}^{l} = (X_{ij1}^{l},..., X_{ijp}^{l})^{T}$ be the vector of covariates, assumed constant over time, for subject *i* in group *j* for transition *l*. Then, we define $T_{ij}^{l} = min(\widetilde {T}_{ij}^{l}, C_{ij}^{l})$, *t*_*ij*_ its realization and $\delta _{ij}^{l} = \mathbf {1}_{(\widetilde {T}_{ij}^{l} \leq C_{ij}^{l})}$. We propose a non-parametric frailty term, which can be modelled through a discrete random variable, *W*^*l*^, with a finite number of masses *K*^*l*^, $w^{l}_{1},...,w^{l}_{K}$, such that $\mathbb {P}\{W^{l}=w^{l}_{k}\}=\pi _{k}^{l}$ for each *k*∈{1,..,*K*^*l*^}. Let $\tilde {\mathbf {w}}^{l}$ be the vector of shared random effects, and **w**^*l*^, $\mathbf {w}^{l}=\exp {\tilde {\mathbf {w}}^{l}}$, be the vector of shared frailties.

We assume that the number of latent populations *K* depends on *l*, so that the number of latent populations detected is transition-specific. In order to build the model, we introduce an auxiliary indicator random variable $z_{jk}^{l}$ which is equal to 1 if the *j*-th group (provider) belongs to the *k*-th latent population in the *l*-th transition, so that $z_{jk}^{l} \overset {i.i.d}{\sim } Bern(\pi _{k}^{l})$. The constraint $\sum _{k=1}^{K} z_{jk}^{l} = 1$, for each *j* and *l*, is equivalent to the assumption that each provider belongs to only one population in each transition.

Then, the conditional hazard function for individual *i* in group *j* for transition *l* is:

1 $$ \lambda^{l}(t;\boldsymbol{X}^{l}_{ij},w^{l}_{k},z^{l}_{jk})=\prod_{k=1}^{K^{l}}\left[\lambda_{0}^{l}(t) w_{k}^{l} \exp((\boldsymbol{X}_{ij}^{l})^{T}\boldsymbol{\beta}^{l})\right]^{z_{jk}^{l}},   $$

where $\lambda _{0}^{l}(t)$ represents the baseline hazard, ***β***^*l*^ is the vector of regression parameters and $w_{k}^{l}$ is the frailty term, shared among providers in the same latent population *k*. Both the frailty and the baseline hazard are assumed to be nonparametric, which makes model (1) a more flexible extension of a proportional hazards Cox model with a shared frailty.

Note that the model for each transition can be interpreted both as a frailty Cox model in which the frailties are shared among members of the same latent population, and also as a population-mixture model, in which each component of the mixture (population) has a different survival distribution, ***π***^*l*^ is the vector of mixing proportions and **w**^*l*^ is the vector of component-specific frailties. Finally, the relative hazard between two individuals with the same covariates, at transition *l*, but from different latent populations *k* and *k*^⋆^ can be described by the frailty ratio $w^{l}_k/w^{l}_{k^{\star }}$.

The unknown parameters in Eq. (1) are $\lambda _{0}^{l}(t)$, ***β***^*l*^, **w**^*l*^, ***π***^*l*^ and *K*^*l*^. All parameters, except for *K*^*l*^, are estimated through a tailored Expectation-Maximization algorithm. For the estimate of *K*^*l*^, the number of latent populations in transition *l*, we cannot use a log-likelihood maximization argument [21]. Consequently, the method proposed by [15] employs a nested algorithm, in which estimation of the parameters $\lambda _{0}^{l}(t)$, ***β***^*l*^, **w**^*l*^, ***π***^*l*^ by the Expectation-Maximization algorithm occurs in an inner loop, while the log-likelihood given a fixed *K*^*l*^ is computed in an outer loop. Each provider is assigned to a specific latent population according to Bayes rule. Then, we estimate *K*^*l*^ according to classical model selection criteria such as Akaike’s Information Criterion (AIC), the Bayesian Information Criterion (BIC), or the approach proposed by [22].

In order to show the outcome of the proposed model and methodology, we provide Cumulative Incidence Functions (CIF) for each transition. The CIF are computed (instead of classical Kaplan-Meier curves) to account for the lack of independence between censoring times and event times, as explained by [23] and [24]. The formula for computing CIF ($\hat {I}^{l}(t_{ij};\boldsymbol {X}^{l}_{ij},w^{l}_{k},z^{l}_{jk})$) is:

2 $$ {\begin{aligned} &\hat{I}^{l}\left(t_{ij};\boldsymbol{X}^{l}_{ij},w^{l}_{k},z^{l}_{jk}\right)\\&\quad= \sum_{r:t_{r} \leq t_{ij}}\hat{\lambda}^{l}\left(t_{r};\boldsymbol{X}^{l}_{ij},w^{l}_{k},z^{l}_{jk}\right)\hat{S}\left(t_{r-1};\boldsymbol{X}^{l}_{ij},w^{l}_{k},z^{l}_{jk}\right),  \end{aligned}}  $$

in which $\hat {\lambda }^{l}(t_{r};\boldsymbol {X}^{l}_{ij},w^{l}_{k},z^{l}_{jk})$ is the hazard rate estimated for transition *l* and $\hat {S}(t_{r-1};\boldsymbol {X}^{l}_{ij},w^{l}_{k},z^{l}_{jk})$ is the Kaplan-Meier estimator. CIF for each healthcare provider are shown in the next Section and each CIF is colored according to the latent population for which it has the highest probability of membership.

### Computational aspects

The algorithm for estimating the parameters of Eq. (1) was implemented in the R package discfrail [25] which has previously only been applied to single time-to-event outcomes.

Since we are interested in investigating the impact of healthcare providers on the different transitions and we are able to estimate the number of latent populations *K*^*l*^ for each transition, we build a matrix of latent population patterns. Specifically, we create a matrix *A* (Assignments) with *J* rows (*J* is the number of healthcare providers) and *L* columns (*L* is the number of possible transitions, 4 in this case). Each element (*j*,*l*) records the latent population to which healthcare provider *j* belongs, in transition *l*. Then, we define the matrix of latent population patterns *C* (Configurations) with $(\prod _{l=1}^{L} K^{l})$ rows and *L* columns. To conclude, we count how many times each configuration (row of matrix *C*) appears in our HF example (matrix *A*).

## Results

A total of 42,871 patients hospitalised for HF with primary discharge between January 1st, 2006 and December 31st, 2007 were identified. The characteristics of the cohort are described in Table 1. As previously described in “Statistical model” section, we modelled each hazard function, *λ*^*l*^(*t*), according to Eq. (1) and we assumed the same regression covariates for all transitions: age, gender, a binary variable which was 1 if the patient had more than three comorbidities and the number of procedures undergone by the patient. The choice of covariates was based on HF literature [26, 27] and the covariate of three comorbidities was created after discussion with cardiologists.

### Patient covariates

We present results obtained by applying the methodology of “Statistical model” section to the HF dataset. A summary of the computed estimates is reported in Table 2 and in each column the transition specific estimates are reported.

**Table 2:** Estimates of the number of latent populations, proportion of hospitals attributed to each population, frailties and hazard ratios for each transition in the HF application. (HR denotes hazard ratio and S.E. denotes standard error

	Cox with nonparametric frailty			
Parameters	Adm. → Disch.	Adm. → Death	Disch. → Adm.	Disch. → Death
	AIC: 5	AIC: 3	AIC: 4	AIC: 3
*K*	BIC: 5	BIC: 3	BIC: 4	BIC: 1
	Laird: 8	Laird: 7	Laird: 7	Laird: 5
	$\pi _{1}^{1} = $ 0.14			
	$\pi _{2}^{1} = $ 0.23	$\pi _{1}^{2} = $ 0.38	$\pi _{1}^{3} = $ 0.18	
***π***	$\pi _{3}^{1} = $ 0.40	$\pi _{2}^{2} = $ 0.47	$\pi _{2}^{3} = $ 0.33	$\pi _{1}^{4} = 1$
	$\pi _{4}^{1} = $ 0.15	$\pi _{3}^{2} = $ 0.15	$\pi _{3}^{3} = $ 0.36	
	$\pi _{5}^{1} = $ 0.08		$\pi _{4}^3 = $ 0.13	
	1			
	1.45	1	1	
frailty ratio	1.76	1.50	1.77	1
	2.27	2.41	2.38	
	2.93		2.91	
HR _*AGE*_ (S.E.)	0.99(4.01 10^−4^)	1.05(1.47 10^−3^)	1.02 (7.40 10^−4^)	1.07(8.92 10^−4^)
HR _*SEX*_ (S.E.)	1.07(8.82 10^−3^)	1.21 (2.50 10^−2^)	1.26 (1.51 10^−2^)	1.24(1.51 10^−2^)
HR _3*COM*_ (S.E.)	0.76 (8.77 10^−3^)	0.88 (2.50 10^−2^)	1.55(1.50 10^−2^)	1.45(1.47 10^−2^)
HR _*NPRO*_ (S.E.)	0.69 (6.56 10^−3^)	0.65(2.29 10^−2^)	0.87(1.15 10^−2^)	0.76 (1.45 10^−2^)

Initially, we note that patient covariate effects were consistent with previous analysis of this dataset [20] and with the literature. For the transition from admission to discharge, older age led to slightly lower instantaneous probability of discharge (estimated hazard ratio 0.99 per year increase in age), corresponding to longer hospital stay, whilst males had a shorter hospital stay (estimated hazard ratio of being discharged 1.07) compared to females. Older patients and male patients had an increased risk of being readmitted to hospital, as well as a higher risk of dying whilst in hospital and out of hospital; results which are consistent with population epidemiology.

Patients who had 3 or more comorbidities had longer stay in hospital (lower instantaneous probability of discharge) and were less likely to die in hospital. The fact that having a higher number of comorbidities was protective against in-hospital death seems counter-intuitive. From descriptive statistics it was clear that people with a high morbidity load were those who were older and had lower number of procedures. The prolonged stay in hospital may indicate that clinicians allocated more resources in order to prepare such patients for discharge (longer LOS, prescribing more drugs, examinations and so on). This fact may also have resulted in lower in-hospital mortality (51.76% of people who died during the first hospitalisation had less than 3 comorbidities). However, patients with more than 3 comorbidities who survived to discharge had higher risk of death outside hospital (fourth column of Table 2) and higher risk of subsequent readmission.

Patients who had a higher number of procedures had a lower instantaneous probability of discharge, corresponding to a longer stay in hospital. However, having a higher number of procedures conferred lower risk of subsequent admissions and death either in or out of hospital. Patients who undergo surgical and other cardiac procedures are highly selected based on their ability to tolerate invasive treatment [28]. The prolonged stay in hospital may reflect the time taken to complete procedures and the need to recover, which will require longer initial stay but with improved health status thereafter.

### Latent populations detection

Focusing on the transition from admission to discharge (column one in Table 2), we note that both AIC and BIC were consistent with a total of 5 latent populations, meaning that 5 time to discharge patterns were detected among the 140 providers. According to the method for model selection proposed by [22], 8 latent populations were identified but this method is known to overestimate the parameter *K*^*l*^, see [15]. Consequently, we focus on the more parsimonious models suggested by the BIC. Latent populations associated with the most extreme frailty values were smaller (for example, providers who were members of populations 1 and 5 with probabilities $\pi _{1}^1 = 14\%$ and $\pi _{5}^{1} = 8\%$ respectively). They correspond to clusters of healthcare providers that were associated with a lower (population 1) or a higher (population 5) risk of transition, after adjusting for patient characteristics. In particular, a patient who was admitted to a healthcare provider from latent population 5 had a higher instantaneous probability of discharge (hazard ratio 2.93) and lower LOS than a patient with the same characteristics (in terms of covariates) who was admitted to a healthcare provider from latent population 1.

Similar patterns were observed for other transitions. In particular, we note that in transition 2, corresponding to in-hospital death (second column of Table 2), three latent populations were detected, with $\pi _{1}^{2} = 38\%$, $\pi _{2}^{2} = 47\%$ and $\pi _{3}^{2} = 15\%$; being admitted to a healthcare provider from latent population 3 conferred increased risk of dying compared to a patient with identical covariates that was admitted to a healthcare provider from latent population 1 (hazard ratio 2.41).

For the discharge to readmission transition (third column of Table 2), the estimated number of latent populations was 4. The proportions associated with the four detected latent populations were: $\pi _{1}^{3} = 18\%$, $\pi _{2}^{3} = 33\%$, $\pi _{3}^{3} = 36\%$ and $\pi _{4}^{3} = 13\%$, with increasing frailty ratios.

Finally, for the transition representing death outside of hospital one latent population was detected (fourth column of Table 2). This might be expected, since there is no reason to connect a death outside hospital to the last healthcare provider from which the patient was discharged. To emphasize this point, the mean (standard deviation) time to death since the last date of discharge was 587 (609) days.

Note again that the term *frailty* in this section is used in its technical sense, to describe a shared underlying rate of an event (in this case, discharge, readmission or death), and this rate is termed a *hazard*.

**Fig. 2:**
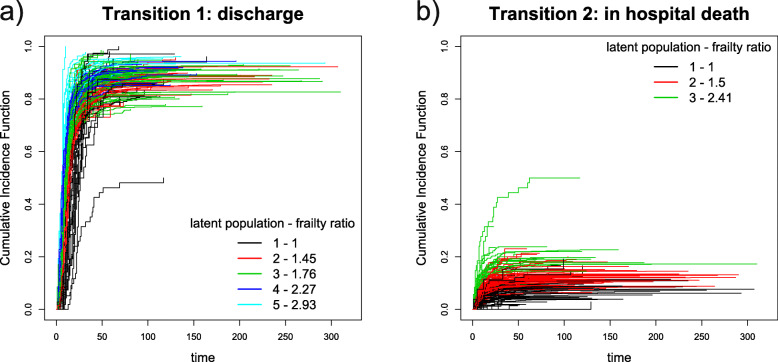
Cumulative Incidence Functions for transition 1 and 2. CIF for transition 1 (admission to discharge) are represented in panel (**a**) and CIF for transition 2 (admission to death) are represented in panel (**b**)

**Fig. 3:**
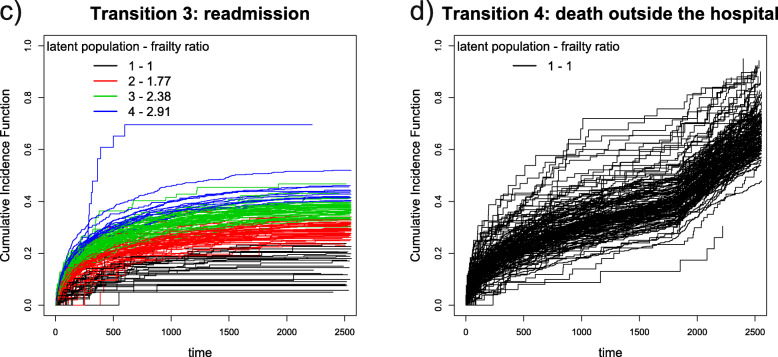
Cumulative Incidence Functions for transition 3 and 4. CIF for transition 3 (discharge to readmission) are represented in panel (**a**) and CIF for transition 4 (discharge to death outside hospital) are represented in panel (**b**)

CIF for each healthcare provider, colored according to the most likely latent population are shown in Fig. 2 for time from hospital admission to discharge (panel (a)) and for time from hospital admission to death (panel (b)). Similarly Fig. 3 shows CIF for time from discharge to the next readmission (panel (a)) and for time from discharge to death outside hospital (panel (b)). Latent populations with the lowest frailty values are labelled with "1" and colored black, while latent populations characterised by the highest frailty values are labelled with " *K*^*l*^" (respectively "5" for discharge, "3" for in-hospital death, see Fig. 2, and "4" for readmission, see Fig. 3).

In almost all transitions we detected the presence of clusters of providers, via the nonparametric discrete frailty in the model. This exploratory tool can be further exploited in two ways: first, we can investigate the characteristics of providers belonging to the same latent population (see [15]); second, we can investigate whether there are specific patterns of latent populations across transitions. In the next subsection we address the second type of analysis.

### Latent populations patterns across transitions

Recalling “Statistical model” section, we count the frequencies of different patterns of latent populations observed in our data.

**Fig. 4:**
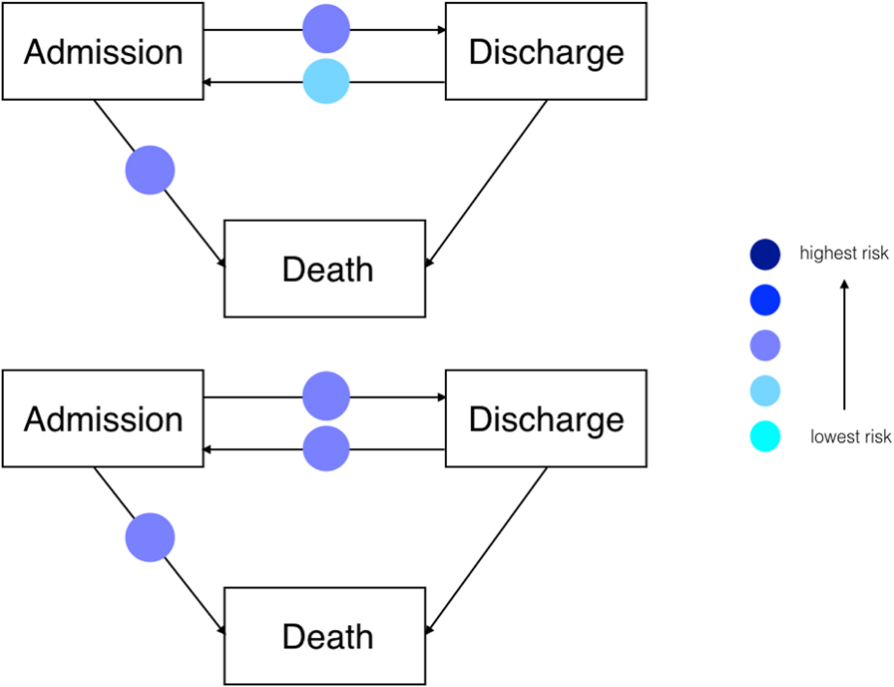
Patterns of latent populations across the four transitions. The upper panel relates to the pattern [3,2,2,1] and the lower panel to [3,2,3,1], the two most common combinations of transition-specific frailty ratios, among hospitals. No color is chosen for Discharge → Death, because only one latent population is identified for this transition

The most common latent population patterns are presented in the rows of Table 3 and the two most common patterns are presented in Fig. 4.

**Table 3:** Under the clustering structure selected by BIC, the commonest combinations of frailty ratios for each transition, and the number (%) of hospitals estimated to have that combination of frailty ratios

Discharge (5 latent populations)	In-hosp. death (3 latent populations)	Readmission (4 latent populations)	Death out (1 latent population)	Number (%)
3	2	2	1	10 (7.1%)
3	2	3	1	10 (7.1%)
3	1	3	1	8 (5.7%)
2	2	3	1	8 (5.7%)
4	2	3	1	8 (5.7%)
1	1	1	1	7 (5%)
1	1	2	1	7 (5%)
3	1	2	1	7 (5%)

Population patterns [3, 2, 2, 1] and [3, 2, 3, 1] occurred 10 times each (7.1% out of 140), which means that, for each pattern, 10 healthcare providers belonged to the same latent population in all transitions. Both patterns represent healthcare providers from latent populations with medium or high transitions rates.

Figure 4 demonstrates that the most represented patterns of latent populations were related to average frailty levels, which corresponds to the fact that the highest mixing proportions were observed for the ’average’ frailty levels (see Table 2). Focusing on the first row of Table 3, we note that: latent population 3 for the admission to discharge transition had frailty ratio of 1.76 (40% of providers); latent population 2 for the in-hospital to death transition had frailty ratio of 1.5 (47% of providers) and latent population 2 for the discharge to readmission transition had frailty ratio of 1.77 (33% of providers).

Among the patterns shown in Table 3 some represent the lowest risks of transitioning, specifically [1, 1, 1, 1] and [1, 1, 2, 1] were both repeated 7 times, (5% of 140) and one reflects patients who are at relatively high risk of making transitions ([4, 2, 3, 1] repeated 8 times, 5.7% of 140).

It would be of interest to further investigate *extreme* latent populations to better understand the reasons for either fast or slow movement between admission and out-of-hospital states. One extreme pattern of latent populations consists of providers with high instantaneous probability of discharge (high frailty for transition 1) and low risk of readmission and death (low frailty for transitions 2,3). Such a provider might be considered to have particularly good performance, and may provide useful insight into good practices. Conversely, a second extreme pattern consists of those providers with low risk of being discharged (small frailty for transition 1) and high risk of readmission and death (high frailty for transitions 2,3). Having adjusted for the main epidemiological factors and markers of disease severity, these providers may suggest additional factors that result in lower performance. In our dataset we identified two providers (1.4*%*) that belonged to the latent populations [5,1,1,1] for transitions 1-4 and five providers (3.6*%*) that belonged to the latent populations [3,1,1,1]. Conversely, there was just one (0.7*%*) provider that belonged to the latent populations [1,2,3,1] and two providers (1.4*%*) that belonged to the latent populations [2,2,4,1]. Due to the small numbers of providers associated with these extreme patterns, identification of risk factors using statistical methods is not likely to be successful. Instead, once providers with these patterns have been identified, detailed local audits and further research may be warranted in order to throw light on the reasons for extreme results.

## Discussion

In this paper we propose a novel statistical model that is an appropriate tool for identifying providers that show extreme behaviour. Specifically, we introduce a flexible model that is particularly suitable for longitudinal, hierarchical time-to-event data in which the existence of different clusters of groups over different transitions is suspected. The proposed semi-Markov framework is general in that any number of health states can be included, with time to a single event [15] included as a special case. This allows us to investigate the healthcare system based on patient characteristics combined with random effects due to providers. Using a discrete clustering technique, we are able to investigate groups of latent populations (i.e., clusters of providers) across transitions representing hospital admission, discharge and death, and to investigate the effect of providers on patients’ movement between these states, adjusting for patient-specific characteristics. This contribution to the literature related to healthcare provider profiling allows identification of those providers characterised by higher or lower risk of moving between different health states. The most powerful aspect of this tool is the fact that it can group providers without the need to pre-specify a pattern of provider characteristics, which is a recognised and unsolved issue in the health economics and decision-making literature [5, 29]. A further contribution of this work is the ability to simultaneously incorporate the whole healthcare path of the patients. This is a substantial advantage compared to the existing literature that largely focuses on a single binary measure of performance through logistic regression modelling [3]. Moreover, it can deal with big databases, such as clinical administrative registries, which are increasingly used to gain insights into healthcare issues [6].

### Implications for policy makers

From a healthcare manager’s point of view, our tool stands as a support for detecting providers that show extreme behaviour and need further investigations. Hospital-level characteristics of these providers can provide valuable information regarding the most efficient use of healthcare resources. Where available, provider specific variables can be included in the models and may identify sources of systematic variation related to providers. Where provider characteristics are not available, the proposed method would be the starting point of further investigation and more detailed data collection.

Results, obtained from fitting this (fairly complex) model can be represented as hazard ratios for time-to-event data and mixing proportions of latent populations, which are accessible, readily interpretable and easily communicated to non-statistical stakeholders. This effort to communicate is enhanced by the figures of “Results” section. Through Fig. 2 and 3, healthcare managers and clinicians can have an immediate idea of the impact of healthcare providers on times to patient readmission, discharge and death. Furthermore, through Fig. 4, it is possible to have a complete overview of providers behaviour across transitions, since the most frequent latent population patterns are displayed. Given these results, it should be easier to identify those healthcare providers that require further investigation.

### Strengths and limitations

Several studies have investigated individual aspects of our approach, such as provider profiling, multi-state models and provider-level frailties. The first contribution of our work is that it addresses all these issues simultaneously. Specifically, an important contribution of this work is the introduction of a multi-state model whose transition rates are modelled through Cox regression with nonparametric discrete frailty for providers, thus allowing for both clustering of providers and adjustment for patient case mix. This novel method allows us to consider provider performance on the basis of the complete patient healthcare experience. The results provide a deeper insight than traditional logistic regression approaches, by taking into account multiple time-to-event data. They improve upon continuous frailty models, in that they are able to identify clusters of providers. Moreover, in our approach, clustering of providers is based on a statistical rule (Bayes rule) that is objective and does not rely on any pre-specified threshold. Finally, the models can be implemented using the freely available *R*-package discfrail.

Implementation of such complex models relies on availability of high quality and complete data. For example, in order to get robust estimates of the transition specific latent populations, we need to have providers with enough patients to allow precise estimation of model parameters (about 20 patients is the minimum, according to previous simulation study). Moreover, the model itself assumes that hazards are transition-specific, so no relationship between frailty across transitions is assumed. This is reasonable as long as providers can be assumed to have different behaviour in different transitions. However, it could be of interest to investigate a joint nonparametric discrete frailty, in order to consider a correlation among frailties in different transitions, although such an extension may not be straightforward.

Another requirement is that the time frame should be sufficiently long for running the analysis. This choice strictly depends on the disease that we are analysing and the model that we want to fit. In this case we observe the selected cohort for 5 years, because we were interested in investigating the complete healthcare path of people suffering from HF, which is a chronic disease. However, if the outcome of interest is simply the first discharge or the first readmission or in-hospital death, a shorter time-frame may be suitable [30].

Proportional hazards for both patient characteristics and frailties are assumed throughout this work. Existing methods for assessing this assumption could be developed, as could methods based on flexible survival models, using splines for both baseline hazards and hazard ratios. However, such extensions would be challenging.

## Conclusions

In this article we provided a novel statistical model, that is an exploratory tool for investigating latent clusters of healthcare providers, according to patients’ clinical history. Specifically, we introduced for the first time a semi-Markov multi-state model with a shared nonparametric discrete frailty term. The inclusion of a shared discrete frailty allowed us to model part of the unspecified heterogeneity related to the hierarchical structure of the data and to identify clusters of providers. We applied the proposed model to a dataset related to Heart Failure patients from Lombardia region. Considering that Heart Failure is a chronic disease characterised by repeated hospitalisations, we investigated readmission, discharge and death as states of the multi-state model. We elaborated effective representations of the model results in order to highlight clusters of providers with similar rates of transition between particular states. This tool can be applied and interpreted by clinical stakeholders that are interested in evaluating providers’ performance, taking into account the complete clinical history of the patients. Note also that this model is highly flexible, since the state-space definition can be tailored according to the disease of interest.

## Data Availability

The data that support the findings of this study are available from Lombardia Region but restrictions apply to the availability of these data, which were used under license for the current study, and so are not publicly available. Data are however available from the authors upon reasonable request and with permission of Lombardia Region.

## Abbreviations

Adm.: Admission
AIC: Akaike’s Information Criterion
BIC: Bayesian Information Criterion
CIF: Cumulative Incidence Function
Comorb.: comorbidities
Disch.: discharge
HF: Heart Failure
HR: Hazard Ratio
ICD-9-CM: International Classification of Disease, 9th revision- Clinical Modification
In-hosp.: In hospital
LOS: Length of Stay
S.D.: Standard Deviation
S.E.: Standard Error
